# No evidence of increased mutations in the germline of a group of British nuclear test veterans

**DOI:** 10.1038/s41598-022-14999-w

**Published:** 2022-07-05

**Authors:** Alexander J. Moorhouse, Martin Scholze, Nicolas Sylvius, Clare Gillham, Christine Rake, Julian Peto, Rhona Anderson, Yuri E. Dubrova

**Affiliations:** 1 Department of Genetics and Genome Biology, University of Leicester, Leicester, LE1 7RH UK; 2 Centre for Health Effects of Radiological and Chemical Agents, Department of Life Sciences, College of Health, Medicine and Life Sciences, Brunel University London, Uxbridge, UB8 3PH UK; 3 Department of Epidemiology and Population Health, London School of Hygiene and Tropical Medicine, Keppel St, London, WC1E 7HT UK; 4 Present Address: School of Cellular and Molecular Medicine, University of Bristol, University Walk, Bristol, BS8 1TD UK

**Keywords:** Mutation, Sequencing, Biophysics, Predictive markers

## Abstract

The potential germline effects of radiation exposure to military veterans present at British nuclear tests in Australia and the South Pacific is of considerable interest. We analyzed germline mutations in 60 families of UK military personnel comprising 30 control and 30 nuclear test veterans (NTV). Using whole-genome sequencing we studied the frequency and spectra of de novo mutations to investigate the transgenerational effect of veterans’ (potential) exposure to radiation at nuclear bomb test sites. We find no elevation in total de novo single nucleotide variants, small insertion-deletions, structural variants or clustered mutations among the offspring of nuclear test veterans compared to those of control personnel. We did observe an elevated occurrence of single base substitution mutations within mutation signature SBS16, due to a subset of NTV offspring. The relevance of this elevation to potential exposure of veteran fathers and, future health risks, require further investigation. Overall, we find no evidence of increased mutations in the germline of a group of British nuclear test veterans. *ISRCTN Registry 17461668.*

## Introduction

Fundamental gaps remain in our understanding of the pattern of mutation induction in humans^1–4^. The limited sensitivity of traditional approaches for monitoring newly arising (de novo) mutations (DNMs) in the human germline and their application to the study of the hereditary effects of radiation in humans have often provided conflicting and inconclusive evidence toward the consequences for the children of exposed parents^5–8^. Continued advances in the throughput and accuracy of whole-genome sequencing (WGS) tools and technologies are providing new insights to the type and distribution, consequences and contributors of de novo germline mutation in humans^9–14^. Current estimates predict between 50 and 100 new mutations per individual per generation which corresponds to a background rate per generation of 1 × 10^–8^ single nucleotide variants (SNVs), the dominant subtype of DNMs^11,15,16^. Exposure to ionizing radiation is known to increase the mutation burden with evidence from animal studies showing elevated DNMs following parental exposure to acute high doses of radiation^3^. For instance, highly significant increases in the incidence of structural variants (SVs), indels and clustered DNMs (1–4 SNVs or clusters of 1–2 SNVs and indels within a few base-pairs of each other) have been reported in mice exposed to 3 Gy^17^. In humans, the frequency of minisatellite mutation has been shown to be significantly elevated for families inhabiting the heavily polluted rural areas of Belarus and Ukraine following the Chernobyl accident^18,19^. Similarly, groups of exposed families living in the vicinity of the Semipalatinsk nuclear test site in Kazakhstan (exposed to nuclear fallout; effective dose > 1 Sv), and Techa River residents (exposed to multiple discharges of radioactive waste), also found significantly elevated mutation rates in the germline of irradiated parents^6,20^.

Radiation exposure to military veterans who participated in the UK’s atmospheric nuclear weapon test and experimental programmes in Australia and the South Pacific in the 1950s and 60 s and, the potential for resulting germline effects, are of considerable interest. In total, ~ 22,000 British troops took part, of which ~ 7000 were alive in 2017. Approximately one quarter of all participants were monitored for external radiation exposure, the majority of these are reported as being negligible and 8% as receiving a ‘non-zero’ dose. Of this 8%, 44 were categorized as receiving between 50 and 100 mSv and 36 of receiving a dose of > 100 mSv. Overall, 759 test veterans were categorized by the Ministry of Defense into ‘special groups’, based upon their role and their potential to receive a higher dose, such as those involved in air plume sampling^21^. Many present at test sites were involved in support roles, such as construction, transport or catering, however additionally, were directly involved with the actual tests, including working in contaminated areas in the days, weeks and months following each test^22^. Such roles may not have been accounted for by the formal categorization into a special group. Additionally, no record of internalized exposure was made.

Epidemiological studies carried out up to 1998 show no evidence of a detectable effect on overall life expectancy or risk of cancer^22^, however this has recently been revised whereby a small excess in mortality (RR = 1.02, 90% CI 1.00–1.05, p = 0.04) associated with similar increased risks for both cancer and non-cancer diseases, compared to non-test veteran controls, is reported^23^. Throughout the intervening decades, questions as to whether veterans could have received sufficient radiation exposure to cause harm and, worry about potential genetic risk of any historical radiation exposure, persist^24,25^. We show elsewhere an over-representation of nuclear test families self-reporting congenital anomalies among their children or grandchildren compared to control families^26^.

The current estimate of human hereditary risk of adverse effect from parental exposure to radiation is 0.2% per Gy. This is based upon extrapolations from animal studies and not through observed increases in human heredity disease^27^. Estimates of the genetic risk for the families of British veterans are unknown, principally due to uncertainties in exposed human populations in general and the uncertainties in dose received, as noted above. To this end, we employed whole genome sequencing tools to examine for any difference in the frequency or spectrum of germline mutations in 30 families of British veterans of nuclear tests (NTV cohort) and 30 families of British military personnel not present at nuclear tests (control cohort).

## Results and discussion

Blood samples were obtained as part of the Genetic and Cytogenetic Family Trio (GCFT) study from the NTV-control family trios of military men (veteran father, mother, child) who were enrolled in the ‘UK nuclear test veterans’ cohort^21^. The sample size of 60 participants was considered sufficient to identify a 1.5-fold increase in the mutation rate as statistically significant (α = 5%, 80% power). The paternal medical diagnostics, parental age at conception, sex ratio among children, number of smokers and levels of alcohol consumption did not differ significantly between the two cohorts (Table S1). To investigate the frequency and spectra of DNMs we performed Illumina paired-end WGS to > 35X coverage to identify de novo SNVs, indels and SVs. The frequency of DNMs and clustered DNMs were investigated and compared between NTV and control families. Details of potential exposure and demographics for sampled family trios, bioinformatic analysis and statistical approach appear in “Materials and methods”.

We first compared our data with those of other larger studies investigating the frequency of DNMs in the general population, our data are in line with these and confirm the effects of parental age on the incidence of DNMs in control families (Fig S1)^13^. We next examined the incidence of transitions and transversions, these are in line with the observations of previous studies and do not significantly differ between the two cohorts (control: 1208 transitions and 643 transversions; NTV: 1193 transitions and 675 transversions; χ^2^ = 0.793; p = 0.37).

**Figure 1:**
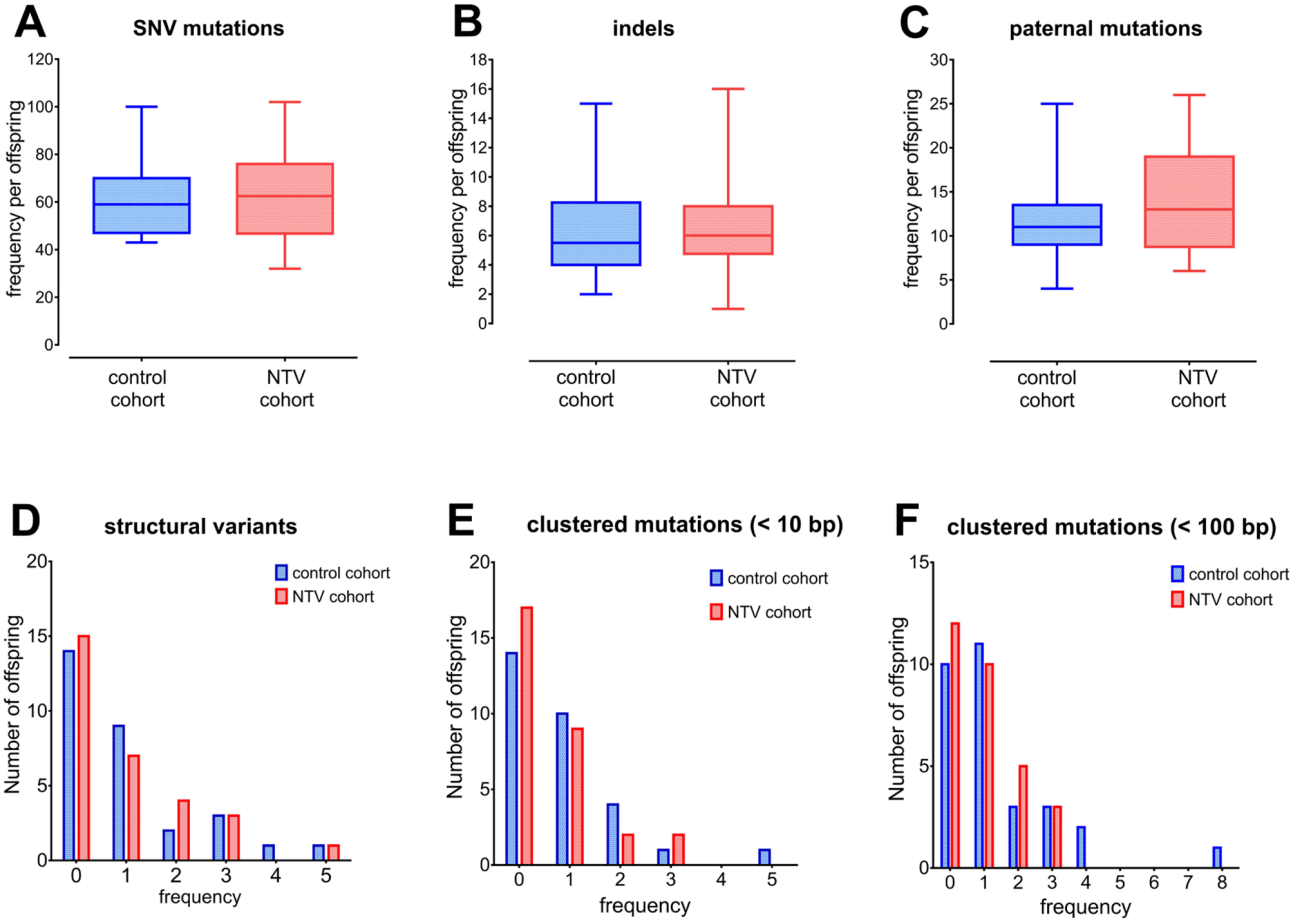
The frequency of de novo mutations per offspring in controls and NTV families. (**A**) SNV mutations; (**B**) indels; (**C**) paternal mutations; (**D**) structural variants; (**E**) clustered mutations (< 10 bp); (**F**) clustered mutations (< 100 bp). 95% confidence intervals are shown.

We next compared the frequency and distribution of DNMs between the NTV and control cohorts, the results of our analysis show no significant differences, and we found no statistically significant differences in the spectra of small indels (< 50 bp) and structural variants (SVs, ≥ 100 bp) between controls and the NTV families (Fig. 1; Table S2A,B). No significant association to any variable indicating a potential radiation exposure was identified for any of the mutation endpoints (negative binomial regression model, Table S3).

**Figure 2:**
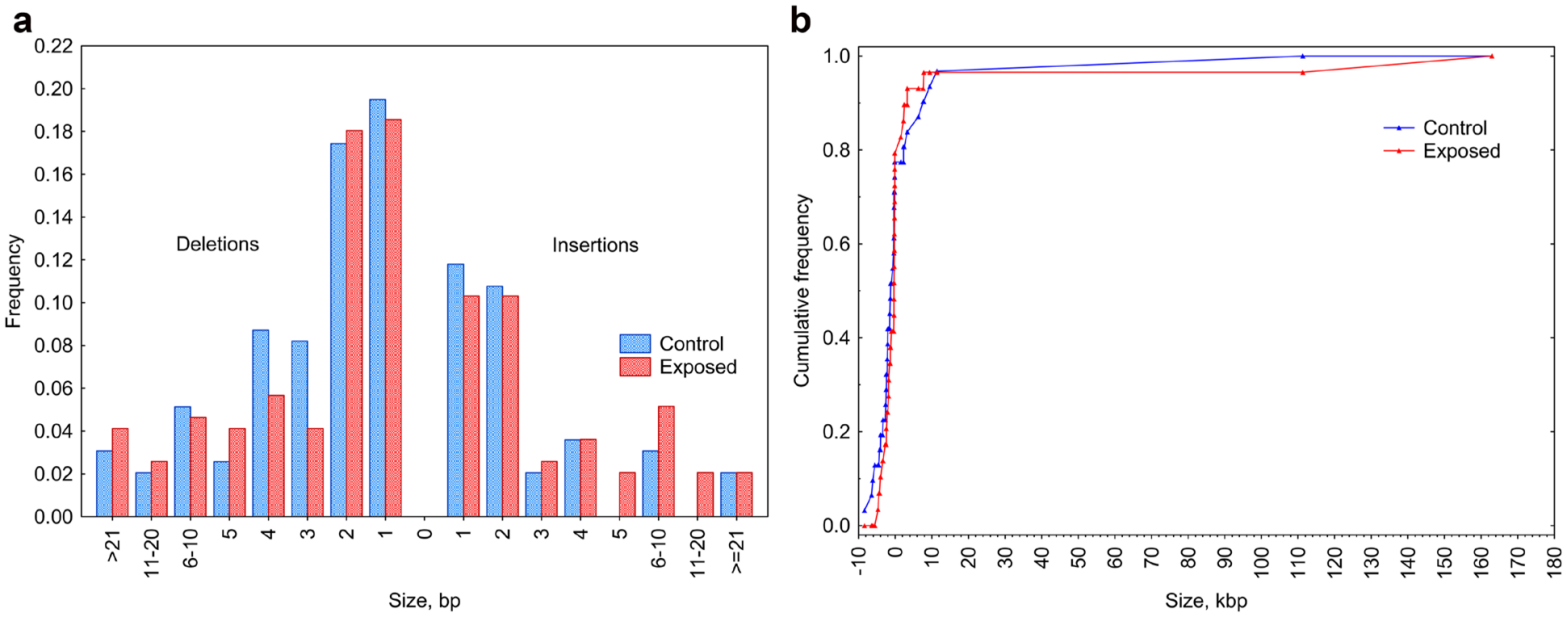
Spectra of indels and structural variants in controls and NTV families. (**A**) The distribution of sizes of de novo indels (Kolmogorov–Smirnov test, *p* = 0.7677); (**B**) The distribution of sizes of de novo structural variants (Kolmogorov–Smirnov test, *p* = 0.5686).

**Figure 3:**
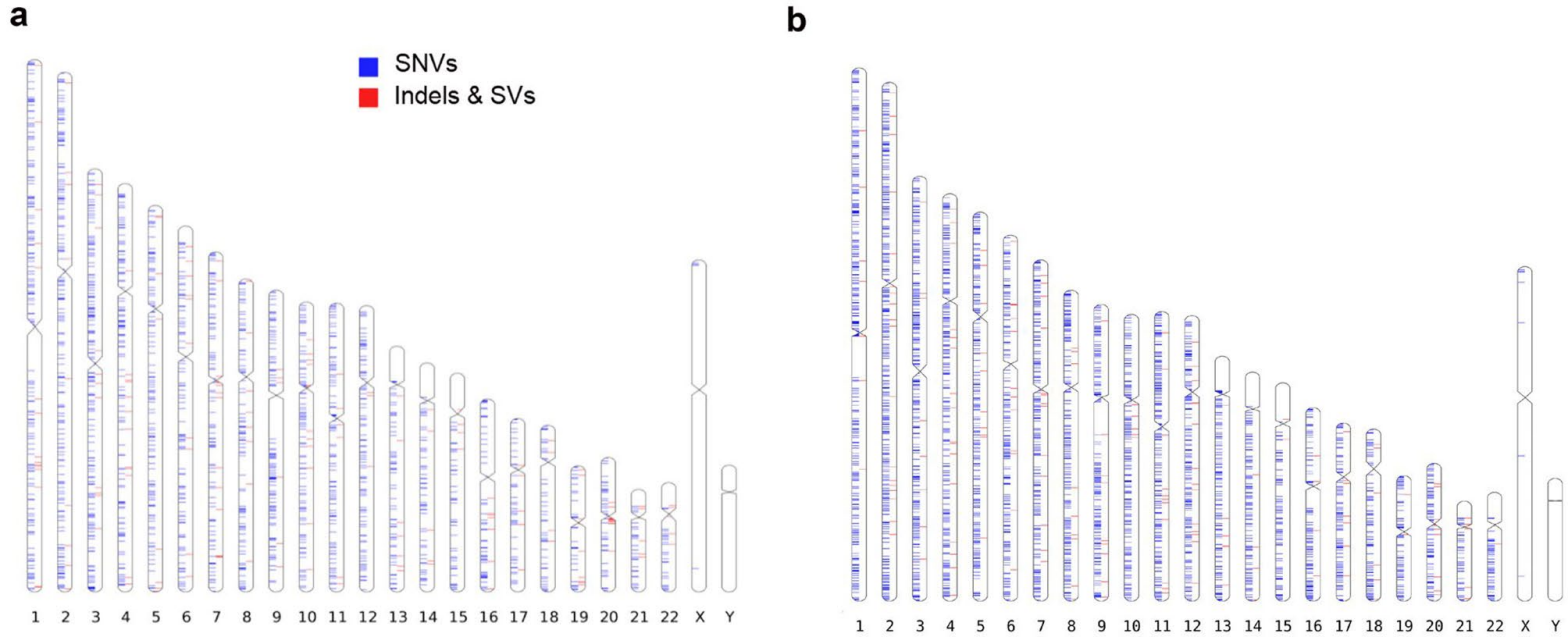
Chromosomal distribution of de novo mutations found in this study. (**A**) control families (**B**) NTV families. Plots drawn by Idiographica.

The incidence of deletions and insertions did not significantly differ (control indels: 130 deletions and 65 insertions; NTV indels: 120 deletions and 74 insertions; χ^2^ = 0.981; p = 0.32; control SVs: 24 deletions and 7 duplications; NTV SVs: 24 deletions and 6 duplications; χ^2^ = 0.06; p = 0.81) (Fig. 2). Clustered mutations are a known feature of exposure to ionising radiation^18^, however in our study we observed no stratification in numbers or types or any significant clustering of induced or spontaneous mutations in control and test families (Fig. 3).

**Figure 4:**
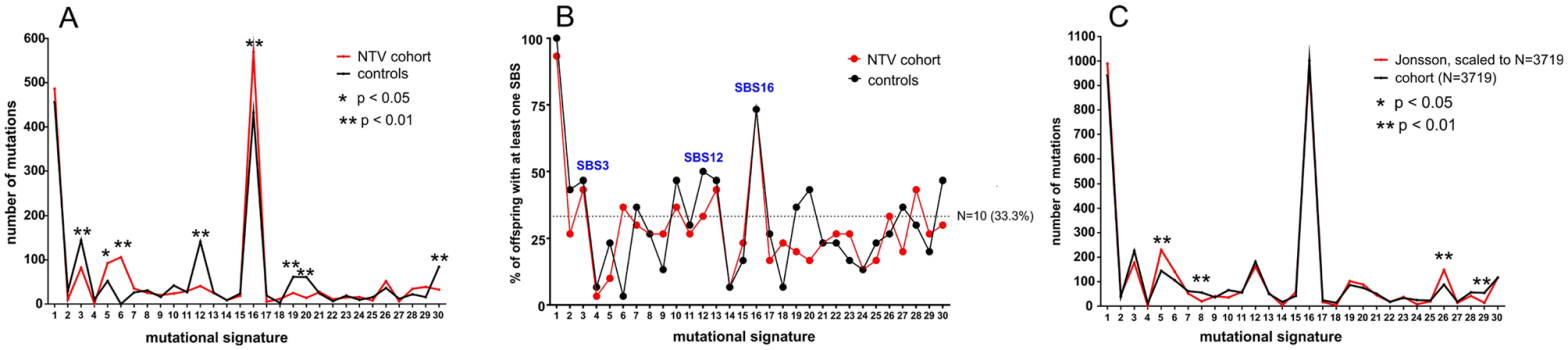
The spectra of mutation signatures in controls and NTV families. (**A**) Frequency of SBS mutations per signature. Data were fitted to the COSMIC v3.2 database with signature fitting distributing each mutation to one (and only one) signature. (**B**). Families (offspring) with at least one mutation per signature. (**C**) SBS signature profiles for the Icelandic cohort and the combined control plus NTV cohorts^11^. Data were fitted to the COSMIC v3.2 database in the same way as the NT and control cohorts, downscaled to the sample size of SNVs identified in our cohorts i.e. N = 3719 in 60 probands. Statistical difference judged on a signature-to-signature basis (Chi square test, multiple p value adjustment by the Holm method).

We next assigned each of the 3719 de novo SNVs identified in control and test families to one of 60 tumour mutation signatures using COSMIC v3.2 (March 2021). This classification is based on the six substitution subtypes: C > A, C > G, C > T, T > A, T > C, and T > G, as well as the nucleotides immediately 5′ and 3′ to the mutation^28^. Two statistical approaches were performed. First, the total number of mutations identified (1851 SNVs in the control cohort and 1868 in the NT cohort) was considered (Fig. 4A). Differences in single base substitution (SBS) mutations from 8 signatures were identified as being statistically significant between the two populations, with signature 16 showing the largest difference (controls: 432, NTV: 569, p < 0.01).

Second, we tested if these differences were still detectable when the average number of SNVs per family (offspring) within each cohort was considered. From these 8 signatures we considered only those appropriate for statistical analysis where at least 10 offspring in each cohort had at least one SNV, resulting into SBS3, SBS12, and SBS16 (Fig. 4B), but neither non-parametric nor parametric approaches could confirm an average difference between control and NTV cohorts (Table S4).

The discrepancy for SBS16 between the two statistical approaches is most likely explained by a subset of families. For instance, when categorising according to the highest number of mutations allocated to SBS16, 5 out of the top 6 occurrences were measured in NT families and corresponded to 40.3% of all mutations for the NTV cohort (n = 30). Thus, a small group in the NTV cohort is mainly responsible for the statistically significant difference of total NTVs in SBS16 signatures identified between both cohorts, however, when the average number of mutations per offspring is considered this significance diminishes (Fig. 4). Therefore, it cannot be ruled out with sufficient confidence that this small group represents a random finding. Of note, the 6 families with the highest number of SBS16 mutations identified here show no indications for associations to any of the confounder variables (child age at sampling, paternal age at conception, occupational radiation exposure (radiologist, nuclear industry, etc.), child and paternal medical diagnostics (X-ray, CT-scan, etc.), but 4 of these families included veterans who had been assigned in the highest rank (rank 3; includes two with a recorded dose of 0.4 and 1.4 mSv) for potential radiation exposure. To date, our understanding of the aetiology of SBS16 mutational signature in normal cells (as sampled here) is largely uncertain but has been attributed to compromised transcription-coupled DNA repair of bulky lesions^28^. Although it is conceivable the finding reported here reflects radiation-induced DNA damage in the test veteran which manifests as the SBS16 signature in offspring, we believe this unlikely given the agreement between our data and those of a larger study of the general population (Fig. 4C)^11,29^.

## Conclusions

A detailed analysis of DNMs in the families of Ukrainian families exposed to the radioactive fallout following the Chernobyl accident has recently been published^14^. Similar to their findings, our study shows no significant increases in the frequency of DNMs in the offspring to nuclear test veteran fathers. Accordingly, the self-reported incidences of congenital and other health effects in descendants are not explained by increases or differences in the spectra of de novo mutations when compared as a group in this study^26^. Further, the effects of de novo mutations on the function of genes were examined (Table S5). Given that we did not find any significant increases in the rate of mutation in the NT group, there is no significant difference in the impact of de novo mutations. Overall, our findings may reflect the very low doses thought to be received by the majority of test veterans. Another consideration is the substantial time gap between the date of last attendance at a test site and conception. The results of studies on cancer patients treated by radiotherapy (al-be-it who receive doses in the order of 10 s of Gys) suggest that these patients undergo a prolonged oligospermia or azoospermia, attributed to cell cycle arrest of the testicular stem cells, during which initial DNA damage can be completely repaired^30^. The observation of an elevated mutation SBS signature 16 in a small number of NT offspring, and the relevance of this finding to future health risks, require further investigation.

## Materials and methods

### Participants

Blood samples were obtained as part of the Genetic and Cytogenetic Family trio (GCFT) study from the NT-control family trios of military men (veteran father, mother, child) who were enrolled in the ‘UK nuclear test veterans’ cohort^21^. This involved gaining information on test veterans who were born 1935 or later, thought to be alive and cancer-free by the custodians of the UK nuclear test veteran cohort (PHE, now UK HSA). Information included service (Royal Airforce (RAF), Navy, Army), location of test site, years attended and any special group status. Special groups included record of dose, noted in Health Physics records and categorization into specialized roles deemed by the Ministry of Defense, UK as having a higher likelihood of exposure such as aircraft handling crew. A total of 5818 veterans were provided, of which only ~ 6% had a record of dose, the majority of which were below 10 mSv (< 1 mSv (293), 1–10 mSv (67), 10–50 mSv (13) and > 50 mSv (4)). Given that only 20% of the entire 22,000 test veterans were issued with a badge and concerns that exposure was not limited to just those issued with film badges, selection was based upon the potential for exposure through attendance at multiple operations and/or allocation into a special group. A long-list of 1459 was generated, which reduced to 908 veterans after flagging with NHS Digital (mainly due to death, diagnosis of cancer or no GP contact detail). Invitations to participate was subsequently carried out in batches with veterans with the highest potential for exposure being prioritized (according to a ranking algorithm identifying those attending multiple tests with special group status). The first 30 complete test veteran family trios who supplied blood where the date of conception of the first child conceived since the last test, was at least 4 months since that date, were included for whole genome sequence analysis^13^.

The 30 control veterans were group-matched on age, service (RAF, Royal Navy, Army) and period of service in tropical regions. Eligible veterans were invited via their GPs and interviewed by telephone using a structured questionnaire to obtain details of service and potential for exposure. Veterans and children were excluded if they ever had cancer (other than non-melanoma skin cancer), or if they were known to have had cytotoxic chemotherapy or radiation treatment for any reason (such as methotrexate for rheumatoid arthritis). Further details of the GCFT study are given by Rake et al.^26^. The GCFT study was conducted in accordance with UK ethical framework and approved by the UK Health Research Authority (17/LO/0273). The NT and control veterans were closely matched by age (median age at blood sample was 80 years in both groups) and service (Table S1). The median age at blood sampling was 76 for NT wives (range 62–84), 77 for control wives (range 63–87), 53 for NT children (range 27–58) and 52 for control children (range 38–60). The median time from exposure to conception among the NT families was 4 years: 16 (53%) 1–4 years, 8 (27%) 5–9 years and 6 (20%) 10–33 years.

### Radiation exposure

The process of verifying participation at nuclear test sites and inclusion into the UK nuclear test veteran’s cohort are described^21,31^. In this study, further verification was carried out revealing very few discrepancies between the details (dates and tests) obtained from the telephone interview and PHE records.

The requirement to obtain blood samples from entire family trios (three family members) limited the recruitment of this already aged population where the veterans were on average aged 80 years old^32^. We were required by the Data Protection Act to invite individuals via their GP practice, which precluded verification of the initial participant invitation as well as the possibility of sending a reminder. The majority of veterans did not reply to the invitation (71% of test veterans and 92% of control veterans) and the proportion of ineligible non-responders was not known. Further to this, the lengthy multi-step nature of recruiting entire family trios (couple GP verification, invitation, screening and consent followed by child invitation via parents, child GP verification, screening and consent and finally blood samples) further reduced the overall trio response rate. Overall, 14% of test and 4% of control families provided at least one blood sample (denominators are taken as number invited excluding those known to be ineligible). From this, 30 test and 30 matched control veterans were analysed to ascertain the frequency and type of germline mutations.

The majority of test veterans in the UK NTV cohort have no recorded dose as not all were issued with film badges and, no measurement for internal contamination took place. For this reason, a proxy for dose was defined. Based on the testimony and verified operation attendance and, blind to any results, the test veterans were assigned to a three-point rank for the potential of internal/external exposure. Each case was a priori assumed to be in the lowest rank (rank 1), and a higher rank allocated only if sufficient information was given to suggest a higher likelihood for radiation exposure. A defined role in a contaminated or forward area (e.g. aircraft sample retrieval/cleaning) undertaken on a repeated basis once was considered a higher exposure potential. Activities which took place immediately and up to 3 months after the test, where dose and dose rates would be expected to be highest were assigned to the higher rank (rank 3) and those which took place at any time from at least 3 months after the test were assigned to the medium rank (rank 2).

Eleven of the 30 case veterans (37%) were assigned to the highest exposure group. Six of these 11 were already identified as members of “special groups” of higher exposed individuals^21^: 3 were aboard the HMS Diana (Montebello) which sailed through plumes and 3 were RAF active handling flight crew (Maralinga and Christmas Island). The remaining 5 veterans with the highest potential for exposure cleaned aircraft or vehicles at Maralinga (n = 4) or supported collecting samples at Christmas Island (n = 1). Subsequent to this ranking, information was linked to recorded doses showing 3 veterans had doses of 0.4 (0.2 on 2 tests), 1.4 and 6.5 mSv. Three veterans were assigned a medium potential for exposure and all accessed forward areas and cleaned aircraft or vehicles but sometime after the tests or less regularly as those assigned the highest rank. The remaining 16 (53%) were assigned the lowest potential for exposure.

### Sequencing

Genomic DNA was extracted from whole blood samples using Qiagen QIAamp DNA Blood Mini kits with the QIAcube automated sample processing instrument according to manufacturer’s instructions. Quality and quantity control of genomic DNA was performed using agarose gel electrophoresis, NanoDrop spectrophotometer and Qubit fluorometer. TruSeq PCR free sequencing libraries were prepared to include a 350 bp insert and were sequenced on Illumina HiSeq instruments for 150 bp paired end reads at ≥ 35 × coverage, ≥ 80% reads at ≥ Q30. Raw data in .fastq format were processed for removal of adapter sequence and low-quality reads and aligned to the human reference genome GRCh38 with alt, decoy and HLA. Alignment and pre-processing was performed using BWA mem, Samtools and Picard. Samples were processed blind in batches until 30 controls and 30 test trios were sequenced and analysed^33,34^.

### Variant analysis

#### SNVs and indels

BAM files were processed for variant detection in batches using Samtools to jointly analyse trio pedigrees for discovery of de novo mutations. Variants were filtered to retain variants with allele frequency < 1% in ALL population from the phase III of the 1000 Genomes Project, variants predicted as deleterious by two or more of four function prediction tools (SIFT, Polyphen, MutationTaster and CADD), variants at exon or splicing regions. This resulted in 13,494 SNVs and 1904 indels. Candidate DNMs were further filtered to remove variants in segmental duplication/repetitive regions, if one or both parents returned > 1 read supporting the alt allele. Coverage depth of ≥ 7 for both parents and ≥ 3 alt reads for the child was required. This resulted in 3386 SNVs and 237 indels candidate DNMs. The Genome Analysis Toolkit (GATK) 4.1.5.0 best practices compliant joint genotyping workflow was implemented once batch processing was complete. Variants that were no longer identified as mendelian violations were removed, resulting in 2964 SNVs and 118 indels. Mendelian violation identified by GATK in cohort mode were additionally retained if the coverage requirements were met as above. Genotype quality (GQ) 99 for the offspring genotype and ≥ 50 for both of the parents were also required. This resulted in 3719 SNVs and 389 indels. These three datasets for each of SNVs and Indels were provided for further analysis^9,35–37^.

*Structural variants* were identified using Delly, Lumpy, Manta, Breaskseq, CNVnator, and SVAba. Detection tools were selected based on performance and implemented as described in comprehensive evaluations of multi-caller detection methods. Vcf files were combined using the overlap-merge approach to derive consensus call sets using SURVIVOR and SVTyper^38–47^. Candidate de novo SVs were retained for further analysis if supported by reads from + and – strands, deletions required support of 6 callers or ≥ 4 callers if ≤ 300 bp for deletions and, duplications required support of ≥ 5 callers or ≥ 4 callers ≤ 1500 bp. Deletions and duplications were removed if not detected in ≥ 2 merge ranges (1000, 500, 250, 150, 50, 25 and 10 bp). This resulted in 163 deletions, 50 duplications, 3 inversions and 2 insertions. Candidate de novo SVs were further discarded if 50 bp merge range yielded de novo SV for more than one pedigree within a 10% range of variant length, this resulted in 22 deletions, and 10 duplications, all with support from ≥ 4 callers, 2 duplications were supported by 5 callers. An alternative final filter for minimum support of 5 callers resulted in 47 deletions and 13 duplications, 38 deletions had support from 6 callers. These three datasets for deletions, duplications, insertions and inversions were provided for further analysis.

*Parental origin* was assigned for SNVs and Indels using GATK PhasebyTransmission and ReadbackedPhasing. Parental origin of structural variants was assigned by visual inspection of reads in Integrated Genome Viewer (IGV)^35,48^.

#### Mutants validation

Primers were designed using a sequence of 400 bp flanking the putative mutation, or break-point in the case of SVs, to amplify a 300- to 425-bp PCR product. Region specific primer sequences were tailed with Illumina compatible sequences and amplicons were prepared using Phusion Flash polymerase in an 8 cycle PCR. Three lanes of balanced pools were sequenced using an Illumina Miseq to yield 250 bp paired end reads at ultra-high depth. Aligned reads were inspected visually in IGV. Of 219 SVs investigated none could be verified as de novo, of 815 SNVs and 156 Indels selected at random, validation rates of 98.1% and 93.8% were obtained, respectively.

### Mutation signature analysis

COSMIC v3.2 (March 2021) database was used as reference for mutational signatures. Reference signature fitting was performed via the Sigflow pipeline, resulting in 30 COSMIC mutational signatures which were considered for further statistical analysis (genome: GRCh38).

### Statistical analysis

The frequencies of mutations in test and control veterans, both overall and in subgroups, were compared by the Kruskal–Wallis test or, if appropriate, Fisher’s exact test, in combination with multiple p value adjustment by the Holm (step-down Bonferroni). To evaluate the influence of potential confounders, the strength of the association between the mutation endpoints, variables for a potential radiation exposure (“exposure” variable) and potential confounder covariates were described by the negative binomial regression model (NB regression). This model was found to be superior to the poison and normal regression model in describing the mutation endpoints (judged by the Akaike information criterion). The NB model is a generalization of Poisson regression and used for modelling overdispersed count variables while adjusting for one or more covariates, and operates on a log link function. Potential covariates in the study were included in the final regression analysis on an endpoint by endpoint basis. In case of highly correlated covariates we selected the one with the most biologically plausible covariate–exposure and covariate–outcome association. The estimated model parameter for radiation exposure is reported as mean and 95% confidence interval, together with its p value, for the unadjusted BM model (i.e. confounder variates are not included in the model) and the adjusted BN model. The statistical mutational SBS signature analysis was supplemented by the Williams version of the binomial regression model (accounting for extra-binomial variation) which used as offspring-specific endpoint the number of SNV mutation allocated to a specific signature out of all SNVs identified in the offspring^49^. All statistical analyses were done using STATISTICA v13.2 (StatSoft Ltd., Bedford, UK) and SAS 9.3 (SAS Institute, Cary NC).

### Ethical approval and consent to participate

The GCFT study was conducted in accordance with UK ethical framework and approved by the UK Health Research Authority (17/LO/0273).

## Supplementary Information

Supplementary Information.

## Data Availability

The dataset generated during this current study are available https://dataview.ncbi.nlm.nih.gov/object/PRJNA788492?reviewer=t65okctpc20o0jfr3n2rmf5n50.
