# Supplementary material for: No evidence of increased mutations in the germline of a group of British nuclear test veterans
Source: Sci Rep. 2022 Jul 5;12:10830. doi: 10.1038/s41598-022-14999-w (PMC9256629; doi:10.1038/s41598-022-14999-w)
Supplement: Supplementary file 1 — Supplementary Information. [file 41598_2022_14999_MOESM1_ESM.docx]

Supplementary Materials for

**No evidence of increased mutations in the germline of a group of British nuclear test veterans.**

Alexander J. Moorhouse^1,2,3^, Martin Scholze^2^, Nicolas Sylvius^1^, Clare Gillham^4^, Christine Rake^4^, Julian Peto^4^, Rhona Anderson^2*^ & Yuri E. Dubrova^1^

Correspondence to: [rhona.anderson@brunel.ac.uk](mailto:rhona.anderson@brunel.ac.uk)

**This PDF file includes:**

Figs. S1

Tables S1 to S5


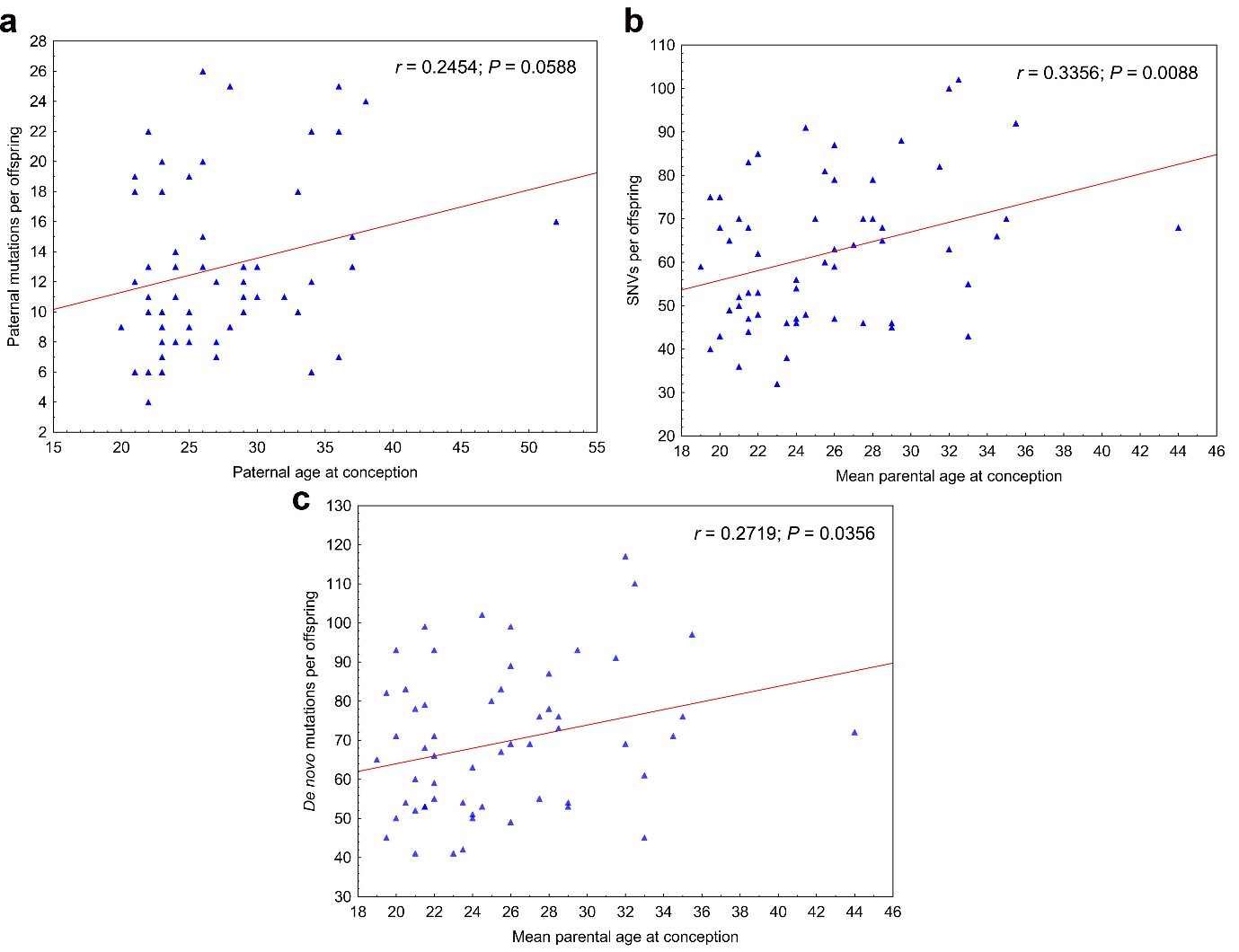


**Fig. S1. Correlation between the age at conception and *de novo* mutations in offspring.** Mean parental age at conception = (paternal age+ maternal age)/2.

**Table S1. Characteristics of study participants**

| Variable | Control (N=30) | NTV (N=30) | Statistics |
| --- | --- | --- | --- |
| Child sex |  |  |  |
| female | 15 | 16 |  |
| male | 15 | 14 | *χ*^2^ = 0.07; *P* = 0.796 |
| Child age at sampling [mean (range) years] | 50.6 (38-60) | 51.6 (27-58) | *t* = -0.64; *P* = 0.522 |
| Child medical diagnostics: number of X-ray | | |  |
| 0 | 9 | 12 |  |
| 1-4 | 9 | 12 |  |
| 5-9 | 3 | 2 |  |
| >10 | 9 | 4 | *χ*^2^ = 2.98; *P* = 0.395 |
| Child medical diagnostics: CT-scan | | |  |
| no | 26 | 26 |  |
| yes | 4 | 4 | - |
| Child medical diagnostic: other scans | | |  |
| no | 24 | 22 |  |
| yes | 6 | 8 | *χ*^2^ = 0.37; *P* = 0,542 |
| Paternal age at sampling [mean (range) years] | 80.0 (76-83) | 79.5 (74-82) | *t* = 1.05; *P* = 0.298 |
| Paternal age at conception [mean (range) years] | 28.1 (21-37) | 26.4 (20-52) | *t* = 1.09; *P* = 0.281 |
| Maternal age at sampling [mean (range) years] | 76.5 (63-87) | 75.8 (62-84) | *t* = 0.57; *P* = 0.571 |
| Maternal age at  conception [mean (range) years] | 24.7 (17-37) | 22.8 (17-36) | *t* = 1.58; *P* = 0.119 |
| Paternal smoking |  |  |  |
| no | 8 | 8 |  |
| yes | 22 | 22 | - |
| Paternal alcohol consumption | |  |  |
| no | 15 | 13 |  |
| yes | 15 | 17 | *χ*^2^ = 0.27; *P* = 0.605 |
| Paternal occupational radiation exposure | | |  |
| no | 19 | 24 |  |
| yes | 11 | 6 | *χ*^2^ = 2.05; *P* = 0.152 |
| Paternal occupational chemical exposure | | |  |
| no | 7 | 14 |  |
| yes | 23 | 16 | *χ*^2^ = 3.59; *P* = 0.058 |
| Paternal medical diagnostics: number of X-ray | | |  |
| 0 | 0 | 1 |  |
| 1-4 | 11 | 13 |  |
| 5-9 | 8 | 7 |  |
| >10 | 11 | 9 | *χ*^2^ = 1.43; *P* = 0.698 |
| Paternal medical diagnostics: CT-scan | |  |  |
| no | 26 | 26 |  |
| yes | 4 | 4 | - |
| Paternal medical diagnostic: other scans | | |  |
| no | 16 | 13 |  |
| yes | 14 | 17 | *χ*^2^ = 0.60 ; *P* = 0.438 |
|  |  |  |  |

t = Satterthwaite t-test statistic; *χ*^2^ = chi-square statistic; - = not applicable

**Table S2A. Number of *de novo* mutations per offspring – cohort differences**

| Variables | % of no-detects | min | P25 | median | P75 | max | Mean | [95% CI] ^1)^ | Mean difference (Kruskal-Wallis test) |
| --- | --- | --- | --- | --- | --- | --- | --- | --- | --- |
| **SNVs** |  |  |  |  |  |  |  |  |  |
| Control | 0 | 43 | 47 | 59 | 70 | 100 | 61.70 | [56.46;67.52] |  |
| NTV | 0 | 32 | 47 | 62.5 | 75 | 102 | 62.27 | [55.91;69.52] | χ^2^ = 0.0342; *P* = 0.8533 |
| **Indels** |  |  |  |  |  |  |  |  |  |
| Control | 0 | 2 | 4 | 5.5 | 8 | 15 | 6.50 | [5.31;7.96] |  |
| NTV | 0 | 1 | 5 | 6 | 8 | 16 | 6.47 | [5.49;7.59] | χ^2^ = 0.0406; *P* = 0.8403 |
| **SVs** |  |  |  |  |  |  |  |  |  |
| Control | 46.7 | 0 | 0 | 1 | 1 | 5 | 1.03 | [0.62;1.74] |  |
| NTV | 50.0 | 0 | 0 | 0 | 2 | 5 | 0.97 | [0.59;1.59] | χ^2^ = 0.0278; *P* = 0.8675 |
| **Total number of mutations** | | |  |  |  |  |  |  |  |
| Control | 0 | 45 | 54 | 68.5 | 78 | 117 | 69.23 | [63.36;75.76] |  |
| NTV | 0 | 41 | 53 | 70 | 83 | 110 | 69.70 | [63.44;76.57] | χ^2^ = 0.0342; *P* = 0.8533 |
| **Clustered <= 10 bp** | | |  |  |  |  |  |  |  |
| Control | 46.7 | 0 | 0 | 1 | 1 | 5 | 0.87 | [0.53;1.4] |  |
| NTV | 56.7 | 0 | 0 | 0 | 1 | 3 | 0.63 | [0.36;1.07] | χ^2^ = 0.6854; *P* = 0.4077 |
| **Clustered <= 100 bp** | | |  |  |  |  |  |  |  |
| Control | 33.3 | 0 | 0 | 1 | 2 | 8 | 1.40 | [0.89;2.21] |  |
| NTV | 40.0 | 0 | 0 | 1 | 2 | 3 | 0.97 | [0.65;1.45] | χ^2^ = 0.5113; *P* = 0.4746 |
| **Paternal mutations** | | |  |  |  |  |  |  |  |
| Control | 0 | 4 | 9 | 11 | 13 | 25 | 12.23 | [10.51;14.25] |  |
| NTV | 0 | 6 | 9 | 13 | 19 | 26 | 13.67 | [11.72;15.97] | χ^2^ = 0.9577; *P* = 0.3278 |
| **Maternal mutations** | | |  |  |  |  |  |  |  |
| Control | 3.3 | 0 | 3 | 4 | 6 | 22 | 4.67 | [3.56;6.16] |  |
| NTV | 0 | 1 | 3 | 4 | 6 | 12 | 4.30 | [3.46;5.32] | χ^2^ = 0.0056; *P* = 0.9405 |
|  |  |  |  |  |  |  |  |  |  |

^1)^ Estimated by profile likelihood function assuming data follow a negative binomial distribution; *χ*^2^ = chi-square statistic

**Table S2B. Number of *de novo* mutations per offspring – Radiation exposure rank^2)^ differences**

| Variables | % of no-detects | min | P25 | median | P75 | max | Mean | [95% CI] ^1)^ | Mean difference (Kruskal-Wallis test) |
| --- | --- | --- | --- | --- | --- | --- | --- | --- | --- |
| **SNVs** |  |  |  |  |  |  |  |  |  |
| Control | 0 | 43 | 47 | 59 | 70 | 100 | 61.70 | [56.46;67.52] | χ^2^ = 0.30; *P* = 0.96 |
| 1 | 0 | 32 | 48 | 62.5 | 70 | 102 | 63.13 | [54.54;73.43] |  |
| 2 | 0 | 46 | 46 | 49 | 79 | 79 | 58.00 | [34.08;109.25] |  |
| 3 | 0 | 36 | 40 | 68 | 79 | 91 | 62.18 | [50.38;77.69] |  |
| **Indels** |  |  |  |  |  |  |  |  |  |
| Control | 0 | 2 | 4 | 5.5 | 8 | 15 | 6.50 | [5.31;7.96] | χ^2^ = 0.94; *P* = 0.82 |
| 1 | 0 | 1 | 5 | 6 | 8 | 15 | 6.44 | [5.05;8.16] |  |
| 2 | 0 | 4 | 4 | 5 | 6 | 6 | 5.00 | [3.95;6.22] |  |
| 3 | 0 | 4 | 4 | 6 | 9 | 16 | 6.91 | [5.15;9.26] |  |
| **SVs** |  |  |  |  |  |  |  |  |  |
| Control | 46.7 | 0 | 0 | 1 | 1 | 5 | 1.03 | [0.62;1.74] | χ^2^ = 0.36; *P* = 0.95 |
| 1 | 56.3 | 0 | 0 | 0 | 1.5 | 5 | 0.94 | [0.41;2.37] |  |
| 2 | 33.3 | 0 | 0 | 1 | 2 | 2 | 1.00 | [0.18;2.98] |  |
| 3 | 45.5 | 0 | 0 | 1 | 2 | 3 | 1.00 | [0.41;2.63] |  |
| **Total number of mutations** | | |  |  |  |  |  |  |  |
| Control | 0 | 45 | 54 | 68.5 | 78 | 117 | 69.23 | [63.36;75.76] | χ^2^ = 0.43; *P* = 0.93 |
| 1 | 0 | 41 | 55 | 70 | 80.5 | 110 | 70.50 | [61.69;80.89] |  |
| 2 | 0 | 51 | 51 | 54 | 87 | 87 | 64.00 | [37.76;120.00] |  |
| 3 | 0 | 41 | 44 | 72 | 89 | 102 | 70.09 | [56.73;87.71] |  |
| **Clustered <= 10 bp** | | |  |  |  |  |  |  |  |
| Control | 46.7 | 0 | 0 | 1 | 1 | 5 | 0.87 | [0.53;1.4] | χ^2^ = 1.47; *P* = 0.69 |
| 1 | 43.8 | 0 | 0 | 1 | 1 | 3 | 0.69 | [0.36;1.17] |  |
| 2 | 66.7 | 0 | 0 | 0 | 1 | 1 | 0.33 | [0.02;1.55] |  |
| 3 | 72.7 | 0 | 0 | 0 | 2 | 3 | 0.64 | [0.19;3.22] |  |
| **Clustered <= 100 bp** | | |  |  |  |  |  |  |  |
| Control | 33.3 | 0 | 0 | 1 | 2 | 8 | 1.40 | [0.89;2.21] | χ^2^ = 1.80; *P* = 0.62 |
| 1 | 31.3 | 0 | 0 | 1 | 1.5 | 3 | 1.00 | [0.59;1.57] |  |
| 2 | 66.7 | 0 | 0 | 0 | 1 | 1 | 0.33 | [0.02;1.55] |  |
| 3 | 45.5 | 0 | 0 | 1 | 2 | 3 | 1.09 | [0.44;2.92] |  |
| **Paternal mutations** | | |  |  |  |  |  |  |  |
| Control | 0 | 4 | 9 | 11 | 13 | 25 | 12.23 | [10.51;14.25] | χ^2^ = 4.93; *P* = 0.18 |
| 1 | 0 | 6 | 9 | 13 | 19 | 24 | 13.81 | [11.16;17.18] |  |
| 2 | 0 | 6 | 6 | 8 | 11 | 11 | 8.33 | [5.81;11.51] |  |
| 3 | 0 | 8 | 9 | 15 | 20 | 26 | 14.91 | [11.51;19.48] |  |
| **Maternal mutations** | | |  |  |  |  |  |  |  |
| Control | 3.3 | 0 | 3 | 4 | 6 | 22 | 4.67 | [3.56;6.16] | χ^2^ = 0.31; *P* = 0.96 |
| 1 | 0 | 1 | 3 | 4 | 6 | 12 | 4.63 | [3.30;6.50] |  |
| 2 | 0 | 2 | 2 | 4 | 5 | 5 | 3.67 | [2.15;5.77] |  |
| 3 | 0 | 1 | 2 | 3 | 6 | 8 | 4.00 | [2.87;5.39] |  |
|  |  |  |  |  |  |  |  |  |  |

1. Estimated by profile likelihood function assuming data follow a negative binomial distribution; *χ*^2^ = chi-square statistic
2. Off spring number per exposure rank: N=16 for Rank=1 (lowest potential for exposure), N=3 for Rank=2 (mid-rank potential for exposure), and N=11 for Rank=3 (highest potential for exposure),

**Table S3: Association between *de novo* mutations per offspring and two variables for a potential radiation exposure (NTV vs. control status, radiation exposure rank)**

|  |  | Unadjusted | |  | Adjusted ^1)^ | |  |
| --- | --- | --- | --- | --- | --- | --- | --- |
| Radiation exposure |  | B [95% CI] ^2)^ | p-value |  | B [95% CI] ^2)^ | p-value |  |
| SNV |  |  |  |  |  |  |  |
| NTV vs. control |  | 0.009 [-0.129; 0.148] | 0.90 |  | 0.017 [-0.109;0.142] | 0.79 |  |
| Exposure rank |  |  |  |  |  |  |  |
| 1 |  | 0.023 [-0.145;0.192] | 0.79 |  | 0.038 [-0.112;0.188] | 0.61 |  |
| 2 |  | -0.062 [-0.387;0.279] | 0.71 |  | -0.017 [-0.324;0.296] | 0.91 |  |
| 3 |  | 0.008 [-0.183;0.202] | 0.94 |  | -0.009 [-0.183;0.167] | 0.92 |  |
| Indel |  |  |  |  |  |  |  |
| NTV vs. control |  | -0.005 [-0.260;0.250] | 0.97 |  | -0.002 [-0.276;0.271] | 0.99 |  |
| Exposure rank |  |  |  |  |  |  |  |
| 1 |  | -0.01 [-0.32;0.298] | 0.95 |  | -0.028 [-0.352;0.294] | 0.86 |  |
| 2 |  | -0.262 [-0.941;0.373] | 0.42 |  | -0.316 [-1.051;0.372] | 0.37 |  |
| 3 |  | 0.061 [-0.287;0.406] | 0.73 |  | 0.100 [-0.279;0.471] | 0.59 |  |
| SV |  |  |  |  |  |  |  |
| NTV vs. control |  | -0.067 [-0.782; 0.647] | 0.85 |  | -0.167 [-0.949; 0.602] | 0.66 |  |
| Exposure rank |  |  |  |  |  |  |  |
| 1 |  | -0.097 [-0.982;0.782] | 0.82 |  | -0.207 [-1.165;0.705] | 0.65 |  |
| 2 |  | -0.033 [-1.828;1.753] | 0.97 |  | -0.081 [-2.030;1.850] | 0.93 |  |
| 3 |  | -0.033 [-1.031;0.964] | 0.95 |  | -0.125 [-1.226;0.964] | 0.81 |  |
| Paternal mutations |  |  |  |  |  |  |  |
| NTV vs. control |  | 0.111 [-0.102;0.324] | 0.30 |  | 0.127 [-0.085;0.339] | 0.23 |  |
| Exposure rank |  |  |  |  |  |  |  |
| 1 |  | 0.121 [-0.128;0.372] | 0.33 |  | 0.148 [-0.099;0.395] | 0.23 |  |
| 2 |  | -0.384 [-0.939;0.158] | 0.16 |  | -0.331 [-0.909;0.222] | 0.24 |  |
| 3 |  | 0.198 [-0.082;0.479] | 0.16 |  | 0.172 [-0.110;0.454] | 0.22 |  |
| Maternal mutations |  |  |  |  |  |  |  |
| NTV vs. control |  | -0.082 [-0.425;0.261] | 0.63 |  | -0.013 [-0.341;0.32] | 0.94 |  |
| Exposure rank |  |  |  |  |  |  |  |
| 1 |  | -0.009 [-0.422;0.405] | 0.97 |  | 0.032 [-0.362;0.426] | 0.87 |  |
| 2 |  | -0.241 [-1.116;0.614] | 0.57 |  | 0.020 [-0.862;0.85] | 0.96 |  |
| 3 |  | -0.154 [-0.64;0.329] | 0.53 |  | -0.088 [-0.560;0.377] | 0.71 |  |
| Clustered <= 10 bp |  |  |  |  |  |  |  |
| NTV vs. control |  | -0.314 [-1.027;0.387] | 0.37 |  | -0.418 [-1.07;0.217] | 0.20 |  |
| Exposure rank |  |  |  |  |  |  |  |
| 1 |  | -0.232 [-1.112;0.605] | 0.59 |  | -0.232 [-1.112;0.605] | 0.59 |  |
| 2 |  | -0.956 [-4.062;0.944] | 0.40 |  | -0.956 [-4.062;0.944] | 0.40 |  |
| 3 |  | -0.309 [-1.366;0.656] | 0.54 |  | -0.309 [-1.366;0.656] | 0.54 |  |
| Clustered <= 100 bp |  |  |  |  |  |  |  |
| NTV vs. control |  | -0.370 [-0.989;0.240] | 0.23 |  | -0.375 [-0.931;0.203] | 0.19 |  |
| Exposure rank |  |  |  |  |  |  |  |
| 1 |  | -0.336 [-1.10;0.399] | 0.37 |  | -0.421 [-1.083;0.242] | 0.21 |  |
| 2 |  | -1.435 [-4.634;0.443] | 0.21 |  | -1.874 [-4.104;0.356] | 0.10 |  |
| 3 |  | -0.249 [-1.113;0.575] | 0.55 |  | -0.092 [-0.842;0.658] | 0.81 |  |
|  |  |  |  |  |  |  |  |

^1)^ adjusted for child age at sampling, paternal age at conception, medical diagnostic radiation exposure (YES/NO), Child medical diagnostics (X-ray, CT-scan, other: YES/NO), paternal medical diagnostics (X-ray, CT-scan, etc.; YES/NO) ; ^2)^ B = unstandardized beta parameter.

**Table S4. Frequencies of SNVs in offspring for S3, S12 and S16 signature**s

| Variables | % of samples with detects | min | P25 | median | P75 | max |  |
| --- | --- | --- | --- | --- | --- | --- | --- |
| S3 |  |  |  |  |  |  |  |
|  |  |  |  |  |  |  |  |
| Control | 46.7% | 0.0 | 0.0 | 0.0 | 5.3 | 38.9 |  |
| NTV | 43.3% | 0.0 | 0.0 | 0.0 | 4.2 | 15.2 |  |
| Mean difference: | | Kruskal-Wallis test | | | p=0.64 |  |  |
|  |  | NB model | | | p=0.31 |  |  |
|  |  | Binomial, overdispersion | | | p=0.17 |  |  |
| S12 | |  |  |  |  |  |  |
|  |  |  |  |  |  |  |  |
| Control | 50.0% | 0.0 | 0.0 | 0.3 | 8.1 | 23.3 |  |
| NTV | 33.3% | 0.0 | 0.0 | 0.0 | 1.7 | 9.5 |  |
| Mean difference: | | Kruskal-Wallis test | | | p=0.11^1)^ |  |  |
|  |  | NB model | | | p=0.07^1)^ |  |  |
|  |  | Binomial, overdispersion | | | p=0.06^1)^ |  |  |
| S16 | | |  |  |  |  |  |
|  |  |  |  |  |  |  |  |
| Control | 73.3% | 0.0 | 0.0 | 10.0 | 24.9 | 57.1 |  |
| NTV | 73.3% | 0.0 | 0.0 | 16.8 | 30.1 | 50.6 |  |
| Mean difference: | | Kruskal-Wallis test | | | p=0.36 |  |  |
|  |  | NB model | | | p=0.49 |  |  |
|  |  | Binomial, overdispersion | | | p=0.21 |  |  |

KW = Kruskal-Wallis test (two-sided); NB = Negative Binomial regression model

^1)^ Bootstrap-adjustment due to small sample size

**Table S5. The impact of *de novo* mutations**

| Mutation sites | Control | NTV |
| --- | --- | --- |
| UTR3 | 25 | 17 |
| UTR5 | 2 | 5 |
| Downstream | 57 | 54 |
| Intergenic | 828 | 856 |
| intronic | 666 | 647 |
| ncRNA exonic | 9 | 17 |
| ncRNA intronic | 167 | 182 |
| Nonsynonymous | 18 | 18 |
| Splicing | 2 | 1 |
| Stopgain | 0 | 2 |
| Synonymous | 10 | 6 |
| *χ*^2^, df=11 | 10.2916 | *P* = 0.5044 |
